# Integrating dissemination and implementation sciences within Clinical and Translational Science Award programs to advance translational research: Recommendations to national and local leaders

**DOI:** 10.1017/cts.2021.815

**Published:** 2021-07-12

**Authors:** Tara G. Mehta, Jane Mahoney, Aaron L. Leppin, Kathleen R. Stevens, Reza Yousefi-Nooraie, Brad H. Pollock, Rachel C. Shelton, Rowena Dolor, Harold Pincus, Sapana Patel, Justin B. Moore

**Affiliations:** 1 Center for Clinical Translational Science, University of Illinois at Chicago, Chicago, IL, USA; 2 The Institute for Clinical and Translational Research, University of Wisconsin, Madison, WI, USA; 3 Center for Clinical and Translational Science, Mayo Clinic, Rochester, MN, USA; 4 National Center for Advancing Translational Sciences, University of Texas Health Science Center, San Antonio, TX, USA; 5 Clinical and Translational Science Institute, University of Rochester, Rochester, NY, USA; 6 Clinical and Translational Science Center, University of California, Davis, CA, USA; 7 Irving Institute for Clinical and Translational Research, Columbia University, New York, NY, USA; 8 Duke Clinical and Translational Science Institute, Duke University School of Medicine, Durham, NC, USA; 9 Clinican and Translational Science Institute & Department of Implementation Science, Division of Public Health Sciences, Wake Forest School of Medicine, Winston-Salem, NC, USA; 10 Mailman School of Public Health, Department of Sociomedical Sciences, Columbia University, New York, NY, USA; 11 New York State Psychiatric Institute and Department of Psychiatry, Columbia University, New York, NY, USA

**Keywords:** Translational science, implementation science, CTSA, workforce, methods, evaluation

## Abstract

The National Center for Advancing Translational Sciences (NCATS) has defined translation as the process of turning observations into interventions that are adopted, sustained, and improve health. Translation must attend to research and community systems and context at multiple levels, and to key stakeholders. Dissemination and implementation (D&I) sciences are informed by an understanding of the critical role of people and systems in disseminating, adopting, and sustaining innovations within real-world settings. Thus, the D&I sciences provides a set of principles that can guide the translational work of Clinical and Translational Science Award (CTSA) programs from basic research to public health. In this special communication, our cross-domain working group of the CTSA consortium, comprised of experts in methods and processes, workforce development, evaluation, stakeholder engagement, and D&I sciences, share a vision of how CTSAs can enhance translation across the translational spectrum through the integration of D&I sciences into the critical areas of methods and processes, workforce development, and evaluation. We propose a set of recommendations for NCATS national and local leaders that are intended to move D&I sciences out of a position of unfamiliarity and ancillary value and into the core identity of who CTSAs are, how they think, and what they do, to advance translation and health.

## Background

The National Center for Advancing Translational Sciences (NCATS) defines translation as the process of turning observations into interventions that are adopted, sustained, and ultimately improve health. Translation must attend to research and community systems and context at multiple levels, along with the key stakeholders within these contexts. Thus, effective and efficient translation depends on the advancement of sciences that can accurately describe and reliably guide relevant processes. Dissemination and implementation (D&I) sciences are informed by an understanding of the critical role of people and systems in disseminating, adopting, and sustaining innovations within real-world settings. As such, D&I sciences provide a set of principles that can guide the translational work of Clinical and Translational Science Award (CTSA) programs.

**Fig. 1.**
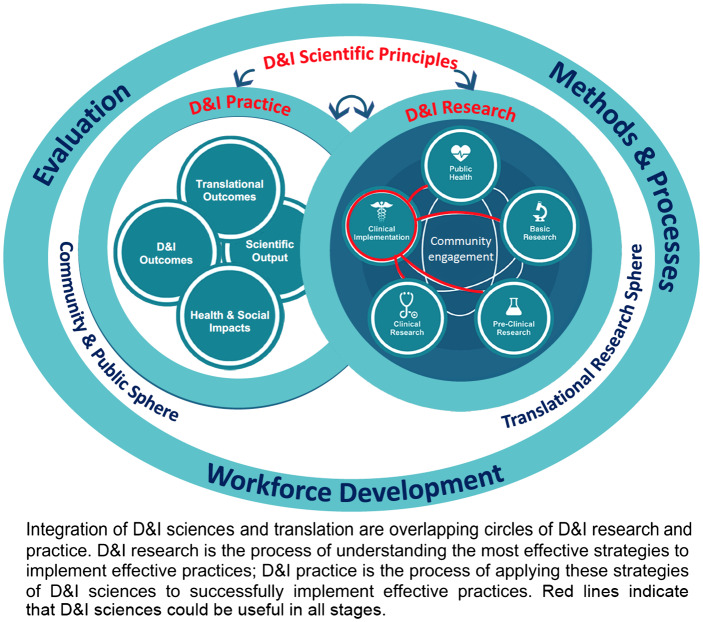
Integrating dissemination & implementation (D&I) research and practice.

Over the past 20 years, D&I sciences have increasingly been applied to promote the late-stage translation of health interventions into diverse health care and community settings, in some cases to address health disparities in care [1]. The application of D&I sciences to late-stage translation often leaves unrealized the positive impact D&I sciences can have on systems and people involved in the earlier stages of translational research (e.g. clinical trialists and other stakeholders involved in research). While the terms “dissemination” and “implementation” are tightly connected to the processes that must occur *after* a viable innovation exists, we posit that the theoretical and methodological principles of D&I sciences apply across the translational science spectrum [2]. In Fig. 1, integration of D&I and translational sciences are presented as overlapping circles of D&I research (i.e. the process of *understanding* the most effective strategies to facilitate the dissemination, implementation, and sustainability of effective practices) and D&I practice (the process of *applying* the most effective strategies to successfully disseminate, implement, and sustain effective practices in real-world settings). While some institutions with CTSAs have embraced the importance of D&I sciences, challenges remain in incorporating D&I sciences into the fabric and infrastructure of CTSAs.

This special communication is the result of discussion by our cross-domain working group of the CTSA consortium, comprised of members of the methods and processes, workforce development, and evaluation workgroups, as well as experts in implementation science and stakeholder engagement, addressing our efforts toward enhancing translation from basic science to public health within our CTSAs via D&I sciences given D&I sciences alignment with translational science [2]. The recommendations encapsulate our experiences to cultivate D&I sciences in CTSAs and common strategies that our group of experts agreed were useful to reach our goal of enhancing the use of D&I sciences in our CTSAs to support translational science. To reach the final set of recommendations, we first presented and discussed our experiences and strategies that we, a panel of experts, had implemented to enhance D&I sciences within our respective CTSAs. Through our discussions, we identified strategies that were often utilized across CTSAs, such as the establishment of a D&I core and D&I consultations and training (e.g. workshops) for trainees and faculty. Finally, we came to a consensus on strategies to present here, which, although not an exhaustive list, were agreed upon as important for integrating D&I sciences into CTSAs and feasible to implement. We propose that these strategies, organized by the domains of methods and processes, workforce development, and evaluation, can be considered suitable for widespread adoption across NCATS and the CTSA consortium (Table 1).

**Table 1.** Recommendations for effective integration of dissemination and implementation (D&I) sciences in Clinical and Translational Science Award (CTSA) programs

Methods and processes
Develop standard expectations and processes for incorporating D&I^1^ expertise and perspectives in CTSA^2^ hub leadership and in key initiatives, methods, and processes.
Advance understanding of different models of D&I^1^ cores and other infrastructures for CTSAs^2^, and methods for collaboration and coordination across centers, including guidance from NCATS^3^ for incorporation into renewal proposals.
Increase involvement of D&I^1^ experts on cross-CTSA initiatives and working groups central to methods and processes, including topics from which they have traditionally been excluded, including clinical trial study design and the responsible conduct of research.
Identify methods by which D&I^1^ sciences can enhance sharing of best practices and programs between CTSA^2^ hubs to promote cross-hub adoption of CTSA^2^ innovations.
Support and track translation of a broader range of innovations into practice, for example, the spread and use of important innovations with high potential for health impact but low market potential.
Evaluation
Develop a set of D&I^1^ competencies for early-stage translational researchers.
Develop D&I^1^ sciences training curriculum for K-scholars, postdoctoral students in translational sciences, doctoral students, and master’s level students.
Identify and catalog novel methods to expand the workforce of D&I^1^ mentors, consultants, and collaborators.
Develop the set of core D&I^1^ competencies to assist partners to engage as scientists, stakeholders, and users of science
Evaluation
Develop novel measures and methods of assessing progress in D&I^1^ advancement and impact within CTSAs^2^, including assessments of faculty D&I^1^ competency, training opportunities and quality, infrastructural and mentorship capacity, methodological alignment with D&I^1^ principles, and translational success.
Establish NCATS-coordinated effort to recruit and train D&I^1^ experts to evaluate CTSAs^2^ with the use of a standardized rubric and approach and a corresponding expectation that D&I^1^ experts should be systematically incorporated into External Advisory Committees and funding review panels
Identify standards for the evaluation of impact resulting from translation of research into practice.

1 D&I: dissemination and implementation

2 CTSA: Clinical and Translational Science Award

3 NCATS: National Center for Advancing Translational Sciences

Our goal is to stimulate interest of D&I sciences for researchers at all stages of the translational spectrum and provide examples demonstrating how D&I sciences can enhance translational science. Furthermore, we hope that our recommendations provide a starting point for CTSA leadership at the university and national level to identify ways to begin integrating D&I sciences into CTSAs. Given the emphasis in D&I sciences on the need for multiple levels of an infrastructure to support change, we contend that our recommendations should be integrated into all levels of CTSAs, from policies to frontline researchers, practitioners and faculty. For example, NCATS could require D&I cores within each CTSA; at the university level, given the science demonstrating that organizational support is critical for change, demonstrated university leadership (e.g. deans) support for D&I sciences within CTSAs is crucial; local university CTSA support might be demonstrated by allocating pilot grant funding focused on D&I sciences. Importantly, this paper points to *what* to do; however, each university will be different in *how* they implement and support D&I sciences within their CTSA due to variation in the local context.

### Implications for Research Methods and Processes

NCATS conceptualizes “methods and processes” as the strategies and approaches that CTSAs and investigators use and advance to improve the efficiency and effectiveness of the conduct of research and research translation [3]. Such approaches include centralized IRBs, agreement on common measures and data elements, strategies for increasing trial recruitment, and study designs and analytic approaches that shorten the time frame for obtaining results. Methods and processes are largely the means to the translational science workforce’s ends. As such, the extent to which methods and processes incorporate the principles and goals of D&I reflects the translational science field’s current perception of D&I’s value. Efforts within CTSAs to promote activities that align with D&I sciences, including “team science,” pragmatic trials, and the rapid movement of research into marketable products, vary by institution but appear to be increasing in recent years [4]. Additionally, community engagement is required of all hubs and is well aligned with stakeholder engagement in implementation science [5]. Some CTSAs have dedicated “D&I cores” or analogous entities (e.g. optional modules) that focus on the late-stage translational research activities of moving health care innovations into practice and/or advancing the sciences of D&I. The D&I cores have made important contributions to our understanding of how to accelerate translational research progress and increase the impact of research products. Yet, even where D&I cores or optional modules exist, the sciences of D&I have not been broadly integrated into the methods and processes of CTSAs, especially in the earliest stages of translational research.

#### Embedding and integrating D&I perspectives

One manner to embed D&I perspectives into current CTSAs is by inventorying existing methods and processes to ensure they benefit from and apply the sciences and principles of D&I. For example, efforts to improve clinical trial recruitment exist in many CTSA hubs, yet, in our experience, frameworks from implementation science that may help inform recruitment strategies are infrequently utilized. An oncologist at Mayo Clinic was recently awarded a career development grant to explore the diverse barriers to clinical trial recruitment and to develop scalable, theory-based strategies for overcoming them, thus serving as an example of the types of opportunities that exist. D&I sciences and principles add value to the methods and processes of collaboration-building, the responsible conduct of research, and D&I practice itself.

Collaboration-building consists of team science and stakeholder engagement activities that bridge the gap between investigators and research users, purveyors, adopters, implementers, policy-makers, and other stakeholders. Successful integration of D&I principles, such as integrated knowledge translation [6] at early stages of the research process, will result in early and intentional convenings of the full range of stakeholders around research and health problems. Specifically, user-centered design [7] could be prioritized to ensure that research-generated solutions are useful, usable, and desirable and that the practice and policy world is prepared to adopt and sustain them.

The responsible conduct of research – including the processes of informed consent, the approval and monitoring of studies, and the ethical inclusion of participants in research – also stands to benefit from D&I sciences. For example, when new approaches for promoting efficient and ethical human subjects protection processes are developed or when new ideas about “appropriate levels of risk” emerge, the use of D&I strategies can promote their spread, uptake, and sustainment.

The value of conducting translational research depends on the selection and use of study designs and evaluation methods that quickly and accurately address study objectives. D&I researchers have expertise in pragmatic effectiveness research, hybrid effectiveness implementation designs, mixed-methods approaches, and the use of diverse methods to explore implementation outcomes relevant to understanding the ultimate generalizability and public health impact potential of health innovations. D&I scientists’ perspectives can be better leveraged, particularly in the design stage of many clinical and translational research projects. CTSAs could also benefit by developing tools and resources to assist investigators in doing research informed by implementation science frameworks, approaches, and methods [8]. Initiatives like the NIH Collaboratory – which function as a learning collaborative to enhance the adoption and effective use of new study designs – serve as an example and model for the CTSA consortium.

D&I practice – literally the practical work of moving research products into real-world use – is where D&I sciences and principles emerged and continue to develop. Although translation of research findings into practice has been a central goal of the CTSA program and many CTSA hubs, funding for the work that is required to prepare innovations for translation into practice is often dwarfed by the funding for basic discovery and early-stage translational research. In the future, CTSAs could expand their funding to support D&I activities necessary for successful translation of innovations into practice, with particular focus on innovations that have high potential health impact but low market potential (e.g. evidence-based psychosocial interventions), as these innovations may lack access to for-profit commercialization channels [9].

#### Building D&I structures

Embedding D&I perspectives into CTSAs will be fostered by an infrastructure that supports integration of D&I sciences within all translational research phases. Requiring CTSAs to establish D&I cores is one way to emphasize D&I’s importance and expand its reach across the translational research spectrum. D&I cores can develop and coordinate pilot funding opportunities to support the design, study, translation, and implementation of health innovations and staff D&I consultants to assist investigators in integrating D&I sciences into research projects that span the translational research spectrum and acquiring external funding for D&I research [10]. Another D&I infrastructure relevant to CTSAs include services for translating and packaging innovations for broad scale-up into practice. For example, the University of Wisconsin-Madison CTSA provides an “Evidence-to-Implementation” award that provides funding and in-kind support from D&I and business experts to develop implementation packages (manuals, train-the-trainer programs) and create business plans to prepare non-patentable innovations for broad scale-up [11]. Lastly, D&I cores can support stakeholder-engaged networks (such as professional societies, local/regional public health entities, mental health providers, and schools) that can facilitate the spread and adoption of research-tested innovations. For example, the Mayo Clinic CTSA organized a regional network of aging services providers to support implementation of evidence-based falls prevention and chronic disease management programs [12, 13]. Finally, because in some cases the need exceeds the capacity of D&I cores to support growing interest in D&I, encouraging cross-CTSA collaboration is critical to facilitate the sharing of resources and information across networks of D&I experts [4].

### Implications for Workforce Development

Integrating D&I sciences within CTSAs will require an increased investment in education and training to ensure that the translational workforce has adequate education in the D&I sciences and access to D&I experts for mentoring and consultation. The need for an increased focus on D&I sciences training and consultation was identified in a survey of CTSA leadership [4]. The survey suggested that awareness of D&I sciences varies widely among translational researchers and only about half of all responding CTSAs (*n* = 20 of 37) directly supported a D&I research or training program, indicating a lack of access to D&I science education and consultation across the CTSA consortium. Respondents identified D&I sciences training activities and access to qualified faculty to lead training and mentorship programs as critical to the ability to develop D&I sciences within CTSAs, underscoring the need for increased investment in access to D&I education and training. Promoting the understanding of D&I principles and how to apply them to enhance research requires identification of critical knowledge and skills (e.g. competencies) and development of effective training strategies [5, 14, 15]. D&I competencies vary by type of workforce; the competencies needed by translational researchers differ from those needed by partners implementing new research into practice.

#### Critical knowledge and skills in D&I sciences

Identifying critical knowledge and skills (e.g. competencies) is complex due to a variety of factors, including (1) different levels of knowledge may be needed at different stages of the translational research spectrum, (2) the types of knowledge that are needed vary by type of workforce (i.e. translational researchers vs. D&I experts vs. stakeholders); and (3) varying levels of knowledge across learner stages. The work of identifying core competencies is in the early stages. Several groups have articulated core competencies for D&I researchers [16]; however, additional work is needed to identify core competencies in D&I sciences that may be relevant to all clinical and translational investigators and to discern which competencies are relevant for particular stages of translation or levels of expertise (e.g. beginner vs. advanced) [17].

We propose four fundamental D&I principles that are important for all translational scientists to understand in order to effectively move their research forward [2] (Table 2). Table 2 provides examples of competencies aligned with each of the four principles, but further work is needed to fully elucidate them in the context of each stage of the translational science spectrum. Currently, the core competencies in clinical and translational research developed by NCATS’ Education Core Competencies Work Group do not include D&I competencies. Adding D&I competencies should enhance translational scientists’ knowledge and skills to better design for dissemination and promote the movement of research along the translational spectrum. For example, The Integrative Framework of Dissemination, Implementation and Translation (IFDIT) describes pathways for multidirectional collaboration between scientists working in the early stages of translational science spectrum and those working in the later stages of D&I [2]. In a recent survey of CTSA Principal Investigators and Administrative Directors, almost two-thirds of respondents reported the need for more training in D&I methods, including in how D&I science can contribute to research across the translational spectrum [4]. Another survey found substantial gaps even among public health researchers in the ability to apply best practices for designing for dissemination [18]. The National Academy of Medicine’s 2017 initiative “*Vital Directions for Health and Health Care”* called for increased patient engagement in product development, use of pragmatic and innovative clinical trial designs, and better identification of product value as three areas essential to speed the uptake of medical advances into clinical practice [19]. D&I sciences address all of these areas. Enhancing translational scientist’s training in D&I sciences should benefit research regardless of the translational research stage (T0 to T4). Specific D&I competencies that are relevant for all research stages include understanding whom to engage as stakeholders in research and how, why, and when to engage them to improve the applicability of research findings, understanding how and why to utilize pragmatic, novel, and efficient study designs and measures, and understanding how to critique the literature with attention to feasibility for translation and impact [2, 20–22].

**Table 2.** Dissemination and implementation science principles and example competencies applicable to Clinical and Translational Science training

Principle	Example competencies to maximize design for ultimate translation
Context matters and is multilevel	• Describe factors that influence research adoption, implementation, maintenance, and reach. • Prioritize questions with high relevance to stakeholders.
It is not sufficient that evidence exists	• Be familiar with user-centered design; making interventions useful, usable, and desirable (design for dissemination). • Understand the stakeholders that should be engaged. • Understand the value of early engagement of stakeholders. • Understand the relevance of study design and choice of target group to external validity and ultimate translatability.
Change happens proactively	• Understand the importance of value proposition, designing for dissemination, cost effectiveness, and policy implications. • Understand the value of type 1 hybrid design in all phases of clinical research. • Understand the sources of error: fidelity/lapses in implementation as a source of reduced/heightened effect.
Both implementation practice and implementation science are team endeavors	• Understand how to identify relevant nonacademic stakeholders in research and how and when to engage with them to aid in movement across research stages and translation into practice. • Understand the benefit of and how to communicate with relevant stakeholders. • Employ weighted evidence, cost-effectiveness, and translation into policy

Within each stage of the translational research spectrum, the degree of mastery of D&I principles that is required varies by level of learner, from masters to PhD to postdoctoral, early stage, and established investigators. The needs of learners change as expertise progresses, from an understanding of how D&I principles may apply, to an understanding of how to apply them to one’s research, to actual application. Graduate students in T0 and T1 research may need basic exposure to factors that affect translatability into practice and the principles underlying design for dissemination. As learners progress toward postdoctoral and K-scholars, greater competency is required. Further work is needed to tailor the training in D&I competencies to fit the level of learner within each stage of the translational research spectrum.

Because not every researcher will be well versed in D&I sciences, it is important that D&I scientists are integrated into teams engaged in early-stage as well as later-stage translational research and have the capacity to effectively consult on early- and late-stage translational research teams. The University of Washington (UW) has developed a novel program to expand the collaborator/consultant workforce in D&I. The University of Wisconsin-Madison utilizes an implementation science monthly discussion group to foster peer learning, peer co-consulting, and networking. Columbia University Irving Institute CTSA provides a consultation program across the university and incorporates a monthly “Works in Progress” meeting where people present grants, papers, and abstracts in progress and get feedback from peers and mentors. Training institutes such as the Training Institute for Dissemination and Implementation Research in Health, Mentored Training for Dissemination and Implementation Research in Cancer, and Implementation Research Institute have helped universities establish a D&I presence, but the demand has outstripped the supply of D&I scientists at many CTSAs [23], demonstrating one ongoing challenge of the consultation. In addition, work is needed to ascertain the effectiveness of D&I consultation models for translational research teams.

#### Scientific workforce training programs

Identifying competencies and principles is a necessary, but not sufficient, condition to develop an effective translational workforce. CTSAs must also support the translation of D&I competencies and principles into action through effective training programs, tailored to specific workforce audiences.

Existing training programs (e.g. KL2, graduate programs, postdoctoral fellowships (e.g. TL1), certificate programs) should be strengthened with the addition of training in D&I competencies and principles. Several strategies for developing and applying competencies in the principles of D&I sciences have been explored and others are being considered across CTSAs [24]. Training in D&I sciences should incorporate sound learning principles, emphasizing both content knowledge and support for initial application of principles and competencies [25]. For example, at the University of Wisconsin-Madison, D&I faculty are piloting “design for dissemination” training and consultations with T2 investigators. The D&I faculty at the University of Wisconsin-Madison CTSA provide a 2 year series consisting of an annual 2 hour seminar plus individual consultation designed to to help K scholars identify and engage with stakeholders and consider D&I within their work. The Year 1 seminar focuses on the value of engaging stakeholders across all translational research stages. University of Wisconsin-Madison is piloting the second phase in which D&I faculty and KL2 directors follow up with interested first-year K-scholars to review goals for stakeholder engagement that align with their research and brainstorm how engaging stakeholders might be helpful, followed by a meeting between the K-scholar and relevant nonacademic stakeholders to discuss translation from the stakeholders’ viewpoint. Finally, an individualized plan for further engagement with stakeholders is designed to understand the aspects related to translation. Some CTSAs include venues beyond the T and K programs for expanding D&I sciences competencies. Examples of professional development programs include national conferences sponsored through CTSAs, including those at the University of Wisconsin-Madison and the University of Colorado Anschutz Medical Campus (Pragmatic Trials) conference (https://coprhcon.learningtimesevents.org/), regional workshops for Texas CTSAs (https://iims.uthscsa.edu/community/activities.html), and increasing D&I programming in the Association for Clinical and Translational Science conferences. Increasingly, the array of available synchronous and recorded online D&I training promotes access to D&I training from thought leaders and experts, ranging from formal programs in implementation science, such as the University of California, San Francisco certificate programs (https://epibiostat.ucsf.edu/certificate-programs), and the University of Colorado D&I certificate program (UC D&I certificate program) to YouTube videos (Training Institute for Dissemination and Implementation Research in Health (TIDIRH, https://obssr.od.nih.gov/training/training-supported-by-the-obssr/training-tidirh/)

#### Development of D&I partners

The translational science workforce consists of investigators working across the translational research spectrum, and individuals who are involved in the implementation and dissemination of innovations in “real-world” settings. Individuals involved in the adoption and implementation of an innovation into practice can include anyone from frontline staff to hospital administrators to policy-makers. In an effort to facilitate the adoption and implementation of innovations within organizations, the role of facilitator (https://www.queri.research.va.gov/training_hubs/behavioral_health.cfm) or knowledge broker [26] is emerging within the D&I workforce.

NCATS is well positioned to increase the capacity of stakeholders to engage in the research process and to advocate for research that is relevant to community settings [27]. This would increase the likelihood of aligning interventions with adopting organizations’ priorities and enhance the potential spread and sustainment of interventions [28, 29]. IFDIT places stakeholder engagement at its center because of the benefits of stakeholder involvement in designing research and implementing the findings [2]. While the benefits of practitioner involvement are clear, including increased likelihood of feasibility and acceptability of an innovation, there remains a paucity of knowledge on effective engagement strategies for particular stakeholder groups [30], and the literature describing research–practice partnerships is emerging [31, 32]. Enabling nonacademic stakeholders to fulfill roles in D&I research will require stated competencies to guide training and a clinical/community environment that encourages engagement in research as a valued part of the work of the clinician/community provider. However, few training programs exist for people in implementation roles (policy-makers, administrators, supervisors, practice improvement facilitators, and frontline clinicians) [23].

In summary, usefully advancing D&I in CTSAs requires equipping the translational workforce – a workforce extending from bench researchers to clinical and community stakeholders – with the competencies needed to apply D&I principles in the relevant contexts.

### Implications for Evaluation of D&I in CTSAs

Currently, we know of no explicit expectations for CTSAs to build D&I capacity nor incorporate D&I research or implementation activities. This is true both for the integration of D&I research expertise and the application of D&I principles to enhance translation across the spectrum. We are not aware of specific measures or activities that are aligned with D&I sciences that CTSAs are expected to implement that will enhance the transition from one translational research stage to another or contribute to the development of D&I knowledge. While some CTSAs support D&I via D&I cores, attempts to assess the impact of D&I cores is challenging. Metrics to assess D&I often include number of grants with a D&I focus, number of supported projects that include a community advisory board, number of investigators that consult with a community advisory board to inform their research, the number of D&I consultations, or the number of grants that include D&I consultants. While such metrics may be useful, they do not capture the broad range of competencies or principles that underlie D&I sciences or whether the competences and principles are integrated into a CTSA’s infrastructure. Additionally, such metrics do not distinguish between the products or processes of the D&I scientist and the application of D&I methods to enhance health care and health outcomes. Lastly, there is no mandate to track adoption, implementation, and scale-up of innovations into practice.

If the recommendations that we propose for the CTSA workforce, methods, and processes are pursued, evaluation efforts should align with recommendations, and measures of impact should be identified, adapted, or created. For example, common and pragmatic measures may be needed to track the acquisition of D&I competencies and skills among faculty across the translational research spectrum, and the application of D&I principles into research practice. Similarly, evaluation of translational science curricula and training programs and of D&I mentorship capacity all follow from efforts to develop the D&I workforce. Evaluation of methods and processes may be more nuanced and will likely benefit from greater inclusion of D&I experts in External Advisory Committees. Innovation is needed to better conceptualize and develop methods and measures of D&I capacity, integration, and impact. Ultimately, if the contribution of better integration of D&I sciences within CTSAs is to be realized, then tracking the impact of CTSA-supported research is essential. One example of a framework that allows for tracking of impact is the Translational Benefits Model [33]. Furthermore, CTSAs must move to documenting and measuring the extent to which research-based innovations are scaled up into practice.

### Conclusion

D&I sciences are intimately connected to translational science, yet poorly integrated and underemphasized in CTSAs. In prior work, our group outlined the rationale for how the application of D&I sciences can advance translational research but acknowledged much effort would be needed to better integrate D&I sciences into the work of CTSAs. The purpose of this paper has been to provide guidance to national and local leaders within our CTSAs (e.g. deans, PIs, faculty, and trainees) to support integration of D&I sciences into CTSAs. Specifically, we propose a set of recommendations that are intended to move D&I sciences out of a position of unfamiliarity and ancillary value and into the core identity of who CTSAs are, how they think, and what they do, to ultimately advance translation and health.

Our recommendations represent the perspectives of a diverse, albeit biased, sample of translational scientists and will benefit from further refinement from an even broader cross section of the field. Engagement of a broader cross section of translational scientists could help define competencies, methods, and processes for investigators across the translational research spectrum to most effectively utilize D&I sciences. In addition, it is important to convene investigators from disciplines that overlap with D&I – such as those from business, marketing, systems engineering, journalism/communication, and social sciences to maximize utility of D&I sciences for CTSAs. Better integration of D&I sciences into the structures and functions of CTSAs will help to advance the public health impact of CTSAs. We trust that CTSAs and NCATS alike will view these thoughts and recommendations as a starting point for careful deliberation and for the laying of plans that will advance translational research and public health.
