# Integrating dissemination and implementation sciences within Clinical and Translational Science Award programs to advance translational research: Recommendations to national and local leaders – ERRATUM

**DOI:** 10.1017/cts.2021.868

**Published:** 2021-12-20

**Authors:** Tara G. Mehta, Jane Mahoney, Aaron L. Leppin, Kathleen R. Stevens, Reza Yousefi-Nooraie, Brad H. Pollock, Rachel C. Shelton, Rowena Dolor, Harold Pincus, Sapana Patel, Justin B. Moore

**Keywords:** Translational science, implementation science, CTSA, workforce, methods, evaluation, erratum

The above article [1] published with an error in one of the headings of Table 1.

The second heading should not read “Evaluation.” The correct heading is “Workforce development.”

The publisher apologizes for this error.
