# Integrating dissemination and implementation sciences within Clinical and Translational Science Award programs to advance translational research: Recommendations to national and local leaders – ADDENDUM

**DOI:** 10.1017/cts.2022.478

**Published:** 2023-04-26

**Authors:** Tara G. Mehta, Jane Mahoney, Aaron L. Leppin, Kathleen R. Stevens, Reza Yousefi-Nooraie, Brad H. Pollock, Rachel C. Shelton, Rowena Dolor, Harold Pincus, Sapana Patel, Justin B Moore

The above article [1] published with missing funding information. In the “Acknowledgments” section of the article, the following text should be added after the first sentence:

This work was also funded in part by the University of Rochester Center for Leading Innovation and Collaboration (CLIC), under Grant U24TR002260. CLIC is the coordinating center for the Clinical and Translational Science Awards (CTSA) Program, funded by the National Center for Advancing Translational Sciences (NCATS) at the National Institutes of Health (NIH).

The authors apologize for this oversight.

The original article has been corrected online to rectify this error.
